# Intracellular Water Indexed to Height Squared as a Complementary Marker to Skeletal Muscle Index Across Nutritional and Hydration Status

**DOI:** 10.3390/muscles5030054

**Published:** 2026-07-29

**Authors:** Yasuhiko Nakao, Masafumi Haraguchi, Fumihiro Mawatari, Ryo Sasaki, Masanori Fukushima, Ryu Sasaki, Satoshi Miuma, Yuko Akazawa, Hisamitsu Miyaaki

**Affiliations:** 1 Department of Gastroenterology and Hepatology, Graduate School of Medicine, Nagasaki University, Nagasaki 852-8501, Japan; 2 Department of Gastroenterology, Kouseikai Hospital, 1-3-12 Hayama, Nagasaki 852-8053, Japan; 3 Neurorehabilitation Research Center, Kio University, Nara 635-0832, Japan; 4 Japan Society for the Promotion of Science, Tokyo 102-0083, Japan; 5 Department of Histopathology and Cell Biology, Graduate School of Medicine, Nagasaki University, Nagasaki 852-8501, Japan

**Keywords:** bioelectrical impedance analysis, skeletal muscle index, intracellular water, body composition, hydration status, sarcopenia, malnutrition, hospitalized patients

## Abstract

Background: Skeletal muscle index (SMI, kg/m^2^) derived from bioelectrical impedance analysis (BIA) is widely used to assess muscle mass. However, SMI is influenced by extracellular fluid, potentially leading to inaccurate estimation in patients with fluid imbalance. Intracellular water normalized by height squared (ICW/height^2^, L/m^2^) may reflect intracellular body cell volume and provide complementary information to BIA-derived SMI, particularly in patients with altered hydration or low nutritional reserve. Based on these considerations, we hypothesised that ICW/height^2^ would demonstrate stronger correlations with BMI than SMI, that the SMI–ICW/height^2^ correlation would differ significantly by sex, and that hydration status (ECW/TBW) would modulate this relationship. Objective: To compare ICW/height^2^ with SMI and evaluate their relationships with nutritional status and hydration in hospitalized patients. Specifically, we tested the hypotheses that (1) ICW/height^2^ correlates more strongly with BMI than SMI; (2) the SMI–ICW/height^2^ correlation differs significantly between sexes; and (3) ECW/TBW and sex interact to modulate the SMI–ICW/height^2^ relationship. Methods: This single-centre, retrospective, exploratory cross-sectional study included 477 hospitalized patients (232 men and 245 women) who underwent body composition assessment using BIA. SMI and ICW/height^2^ were examined in relation to body mass index (BMI) and hydration status assessed by extracellular water to total body water ratio (ECW/TBW). Correlation analyses were performed overall and stratified by sex, BMI categories (<18.5, 18.5–24.9, ≥25 kg/m^2^), and ECW/TBW groups (<0.36, 0.36–0.40, >0.40). Results: ICW/height^2^ showed a stronger correlation with BMI than SMI in both men (R^2^ = 0.492 vs. 0.338) and women (R^2^ = 0.354 vs. 0.121). A strong correlation between SMI and ICW/height^2^ was observed in men (R^2^ = 0.634), whereas the correlation was weaker in women (R^2^ = 0.316). In BMI-stratified analyses, the correlation between SMI and ICW/height^2^ increased with higher BMI in men (R^2^ = 0.192, 0.467, 0.791), while in women it was markedly attenuated in the low BMI group (R^2^ = 0.134). Stratification by ECW/TBW showed that in men, the correlation remained strong under normal hydration conditions but was attenuated in the high ECW/TBW group. In contrast, in women, the correlation was weak even within the normal ECW/TBW range (R^2^ = 0.336) and particularly poor in the low ECW/TBW group (R^2^ = 0.060). Among women with normal ECW/TBW, those with low BMI demonstrated clear discordance between SMI and ICW/height^2^. Exploratory four-quadrant classification using sex-specific SMI and ICW/height^2^ thresholds further demonstrated that patients with discordant SMI and ICW/height^2^ values had distinct BMI profiles, and BMI differed significantly among the four groups in both men and women (Kruskal–Wallis test, *p* < 0.001). Conclusions: These exploratory findings suggest that ICW/height^2^ and BIA-derived SMI may show systematic discordance under specific clinical conditions, particularly in underweight or fluid-imbalanced hospitalised patients. Prospective validation against reference-standard body-composition measures is required before any clinical application.

## 1. Introduction

Assessment of skeletal muscle mass is essential in the diagnosis of sarcopenia and nutritional evaluation in both outpatient and inpatient settings. Imaging modalities such as computed tomography (CT), magnetic resonance imaging (MRI), and dual-energy X-ray absorptiometry (DEXA) are recommended as reference standards [1,2]. However, in routine clinical practice, bioelectrical impedance analysis (BIA) is more commonly used due to its convenience and non-invasiveness [3].

A major limitation of BIA-derived skeletal muscle index (SMI) is its susceptibility to fluid imbalance. In particular, extracellular fluid expansion can lead to potential overestimation of BIA-derived skeletal muscle mass estimates, reducing their reliability in patients with edema or altered hydration status [3,4,5].

Intracellular water (ICW) reflects the volume of water within cells and is closely associated with body cell mass, particularly skeletal muscle cells [6,7,8,9]. Therefore, ICW normalized by height squared (ICW/height^2^) may provide a potentially useful indicator of intracellular body cell volume that is less influenced by extracellular fluid changes. ICW is fundamentally linked to body cell mass (BCM), the metabolically active cellular component of lean body mass; since skeletal muscle constitutes the principal BCM component, ICW normalised by height squared may more directly reflect functional intracellular muscle cell volume than BIA-derived SMI, which incorporates both intra- and extracellular water compartments and is therefore susceptible to extracellular fluid shifts [4,5,6,7,8,9].

Importantly, whether ICW/height^2^ merely parallels SMI or provides additional information on intracellular body cell volume remains unclear. The aim of this study was to evaluate whether ICW/height^2^ is a complementary marker of muscle mass compared to SMI and to determine whether discrepancies between these indices emerge under specific clinical conditions such as altered nutritional status and hydration.

However, the knowledge gap remains: whether ICW/height^2^ and SMI capture distinct aspects of body composition under conditions of altered hydration or nutritional depletion, and whether their relationship differs by sex, has not been systematically examined in hospitalised patients. We therefore formulated three pre-specified hypotheses: (1) ICW/height^2^ correlates more strongly with BMI than SMI, suggesting greater sensitivity to nutritional reserve; (2) the SMI–ICW/height^2^ correlation is significantly weaker in women than in men, reflecting sex-specific body composition characteristics; and (3) ECW/TBW and sex interact to modulate the SMI–ICW/height^2^ relationship, such that the association between hydration status and ICW/height^2^ is stronger in women. The present study was designed to test these hypotheses in a retrospective cohort of consecutively hospitalised patients at nutritional risk.

## 2. Methods

### 2.1. Study Design and Participants

This single-centre, retrospective, exploratory cross-sectional study enrolled consecutive adult patients admitted to Juzenkai Hospital (Nagasaki, Japan) between April 2022 and March 2025. All admitted patients underwent nutritional screening at admission using the Subjective Global Assessment (SGA). Patients identified as being at nutritional risk subsequently underwent body composition assessment using BIA for malnutrition diagnosis and severity grading, in accordance with the institutional Nutrition Support Team (NST) protocol. Where multiple BIA measurements were available during admission, only the first measurement was used. Patients were recruited primarily from Ward 3 (rehabilitation ward, *n* = 196, 41.1%) and Ward 4 (general internal medicine and respiratory medicine ward, *n* = 260, 54.5%); the remaining 21 patients (4.4%) were admitted to other wards. Patients were excluded if height, weight, SMI, ICW, or ECW/TBW data were missing, or if the BIA measurement was considered unreliable (device error or incomplete measurement). Patients with implanted cardiac devices (e.g., pacemakers) or limb amputation, for whom standard BIA is contraindicated, were also excluded. As this was a retrospective, cross-sectional, exploratory study, no a priori sample size calculation was performed; the sample size reflects all consecutive eligible patients during the pre-specified study period. The final analysis included 477 patients (232 men, 245 women).

### 2.2. Measurements

Body composition parameters were obtained using a multifrequency bioelectrical impedance analysis device (InBody S10; Biospace Co., Ltd., Seoul, Republic of Korea). A single InBody S10 unit was available at the institution; all measurements were performed in the supine position in the morning, before meals, and after urination where possible.

- Skeletal muscle index (SMI, kg/m^2^);
- Intracellular water normalized by height squared (ICW/height^2^, L/m^2^);
- Body mass index (BMI, kg/m^2^);
- Extracellular water to total body water ratio (ECW/TBW).
- 50 kHz whole-body phase angle (°) [10].

### 2.3. Stratification

Patients were categorized according to BMI:

- BMI < 18.5;
- 18.5 ≤ BMI < 25;
- BMI ≥ 25.

Hydration status was classified as:

- Low (<0.36);
- Normal (0.36–0.40);
- ECW/TBW > 0.40 was used to define high hydration status based on published BIA reference ranges and prior studies using ECW/TBW as an indicator of fluid overload [4,5]; specifically, Okamoto et al. [5] used this threshold to identify post-operative fluid retention. The normal range (0.360–0.400) is consistent with manufacturer reference values for healthy adults. The ECW/TBW < 0.36 threshold was applied as an exploratory lower boundary; this threshold is less established in the published literature and results from this subgroup (*n* = 33, 6.9%) should be interpreted with particular caution. ECW/TBW-stratified analyses and the four-quadrant classification were designated as exploratory; the primary pre-specified analyses were sex-stratified Pearson correlations and multivariable regression.

### 2.4. Exploratory Four-Quadrant Classification

For exploratory analysis, patients were classified into four groups according to sex-specific SMI cut-off values and corresponding ICW/height^2^ thresholds. The SMI cut-off values were 7.0 kg/m^2^ for men and 5.7 kg/m^2^ for women, consistent with the Asian Working Group for Sarcopenia 2019 diagnostic criteria [2], which have been validated in Japanese populations [11]. Low SMI was defined as SMI below these thresholds. Note that sarcopenia diagnosis per AWGS guidelines also requires assessment of muscle strength and/or physical performance, which were not systematically collected in this study; therefore, the term “low SMI” is used throughout in place of “sarcopenia.” The provisional ICW/height^2^ thresholds were 7.46 L/m^2^ for men and 6.84 L/m^2^ for women. Four groups were defined as follows: both SMI and ICW/height^2^ above the thresholds, low SMI with preserved ICW/height^2^, preserved SMI with low ICW/height^2^, and both SMI and ICW/height^2^ below the thresholds.

### 2.5. Statistical Analysis

Continuous variables are presented as mean ± standard deviation. Correlation analyses between variables were performed using Pearson’s correlation coefficient, and coefficients of determination (R^2^) were calculated. Subgroup analyses were conducted based on BMI and ECW/TBW categories. Comparisons of BMI among the four exploratory SMI/ICW groups were performed using the Kruskal–Wallis test. Statistical analyses were performed using R (version 4.3.3; R Foundation for Statistical Computing, Vienna, Austria). Pearson’s correlation coefficients and 95% confidence intervals were calculated using Fisher’s z-transformation. Group comparisons across the four exploratory classification groups were performed using the Kruskal–Wallis test. A two-sided *p* value of <0.05 was considered statistically significant. The primary analyses were the overall and sex-stratified Pearson correlations between SMI and ICW/height^2^, between BMI and each index, and multivariable regression. All subgroup analyses stratified by BMI or ECW/TBW category, the sex × ECW/TBW interaction model, and the four-quadrant classification were designated as exploratory. The distribution of key continuous variables was examined using skewness and excess kurtosis prior to correlation analyses: SMI showed moderate positive skewness (0.86), ICW/height^2^ demonstrated pronounced right-skewness (skewness 1.50, excess kurtosis 7.57), and ECW/TBW showed left-skewness (−1.27). Because Pearson’s correlation may be influenced by skewed distributions and outliers, Spearman’s rank correlation was additionally computed as a sensitivity analysis, yielding directionally consistent results (ρ_overall = 0.740, ρ_men = 0.761, ρ_women = 0.702). The sex difference in correlation coefficients between SMI and ICW/height^2^ was formally tested using Fisher’s z-transformation. No a priori power calculation was performed, as this was a retrospective, exploratory study without pre-specified effect sizes.

## 3. Results

### 3.1. Patient Characteristics

A total of 477 patients were included in the analysis, comprising 232 men and 245 women. The mean age was 76.0 ± 14.1 years in men and 82.9 ± 13.2 years in women. The mean body mass index (BMI) was 21.1 ± 4.3 kg/m^2^ in men and 19.8 ± 3.8 kg/m^2^ in women. The mean skeletal muscle index (SMI) was 6.5 ± 1.4 kg/m^2^ in men and 5.0 ± 1.4 kg/m^2^ in women. The mean intracellular water normalized by height squared (ICW/height^2^) was 6.9 ± 1.4 L/m^2^ in men and 6.0 ± 0.9 L/m^2^ in women. Disease groups included: internal medicine (*n* = 138, 28.9%), cerebrovascular disease (*n* = 122, 25.6%), respiratory disease (*n* = 68, 14.3%), cancer (*n* = 37, 7.8%), musculoskeletal disease (*n* = 35, 7.3%), urological disease (*n* = 29, 6.1%), cardiovascular disease (*n* = 25, 5.2%), and disuse syndrome (*n* = 23, 4.8%). Patients were primarily admitted to Ward 3 (rehabilitation ward; *n* = 196, 41.1%) and Ward 4 (general internal medicine/respiratory ward; *n* = 260, 54.5%); the remaining 21 patients (4.4%) were admitted to other wards. Barthel Index at admission: median 60 (IQR 5–95); 192 patients (40.3%) had BI ≤ 40, indicating severe functional impairment. Hospitalization duration: median 27 days (IQR 11–47).

### 3.2. Correlation Between BMI and Muscle Indices

**Figure 1:**
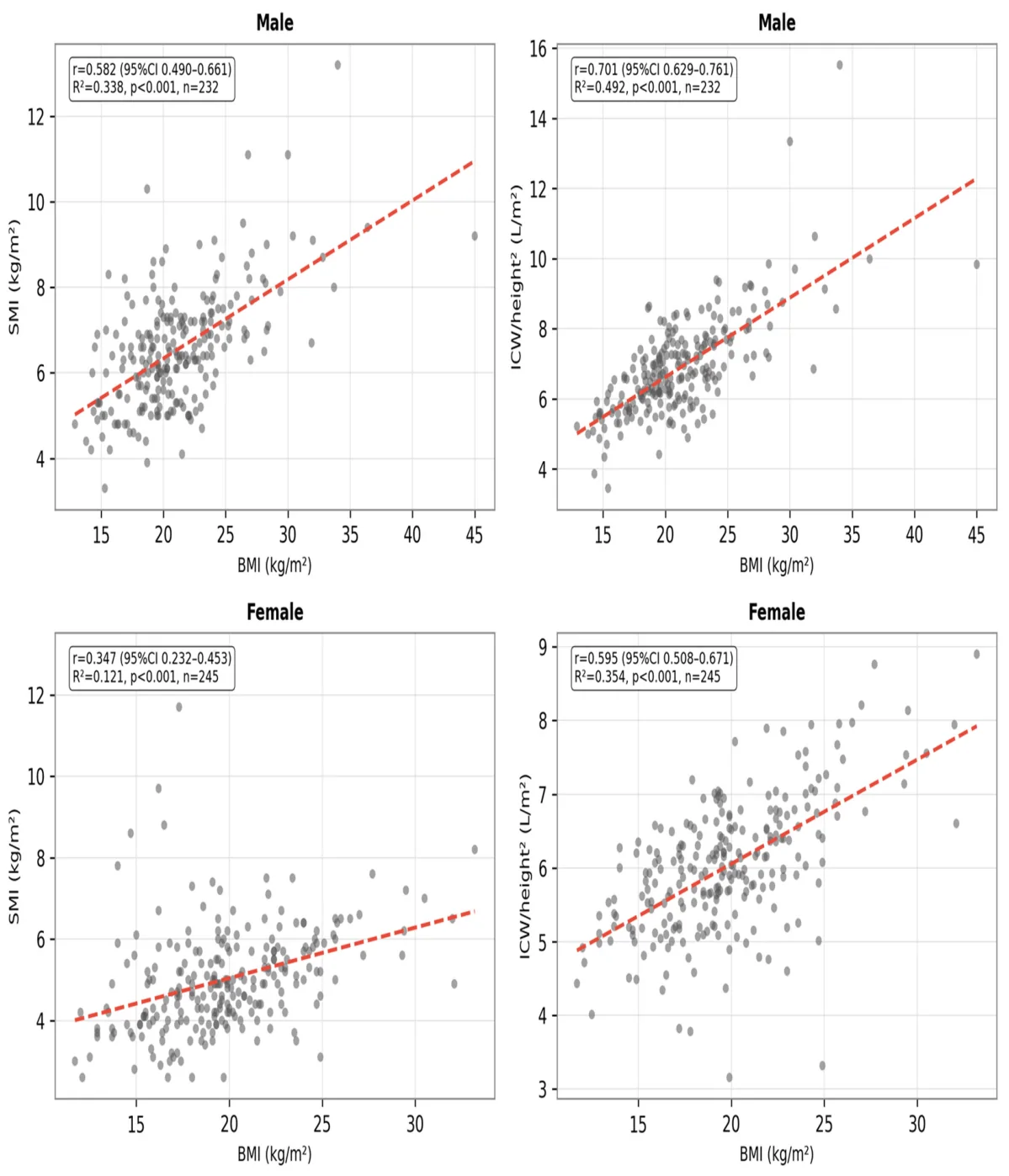
Relationship between BMI and muscle indices. Scatter plots showing Pearson correlations between BMI and SMI (**left column**) and between BMI and ICW/height^2^ (**right column**), stratified by sex. Regression lines, r with 95% CI, R^2^, and *p* values are shown. ICW/height^2^ showed consistently stronger correlations with BMI than SMI in both men (R^2^ = 0.492 vs. 0.338) and women (R^2^ = 0.354 vs. 0.121).

BMI was positively correlated with both SMI and ICW/height^2^ in both sexes; however, ICW/height^2^ consistently demonstrated stronger correlations with BMI than SMI. In men (*n* = 232), the Pearson correlation between BMI and ICW/height^2^ was r = 0.701 (95% CI 0.629–0.761, R^2^ = 0.492, *p* < 0.001), compared to r = 0.582 (95% CI 0.490–0.661, R^2^ = 0.338, *p* < 0.001) for BMI and SMI. In women (*n* = 245), the difference was more pronounced: BMI vs. ICW/height^2^ yielded r = 0.595 (95% CI 0.508–0.671, R^2^ = 0.354, *p* < 0.001), whereas BMI vs. SMI yielded r = 0.347 (95% CI 0.232–0.453, R^2^ = 0.121, *p* < 0.001) (Figure 1).

### 3.3. Association Between SMI and ICW/Height^2^

**Figure 2:**
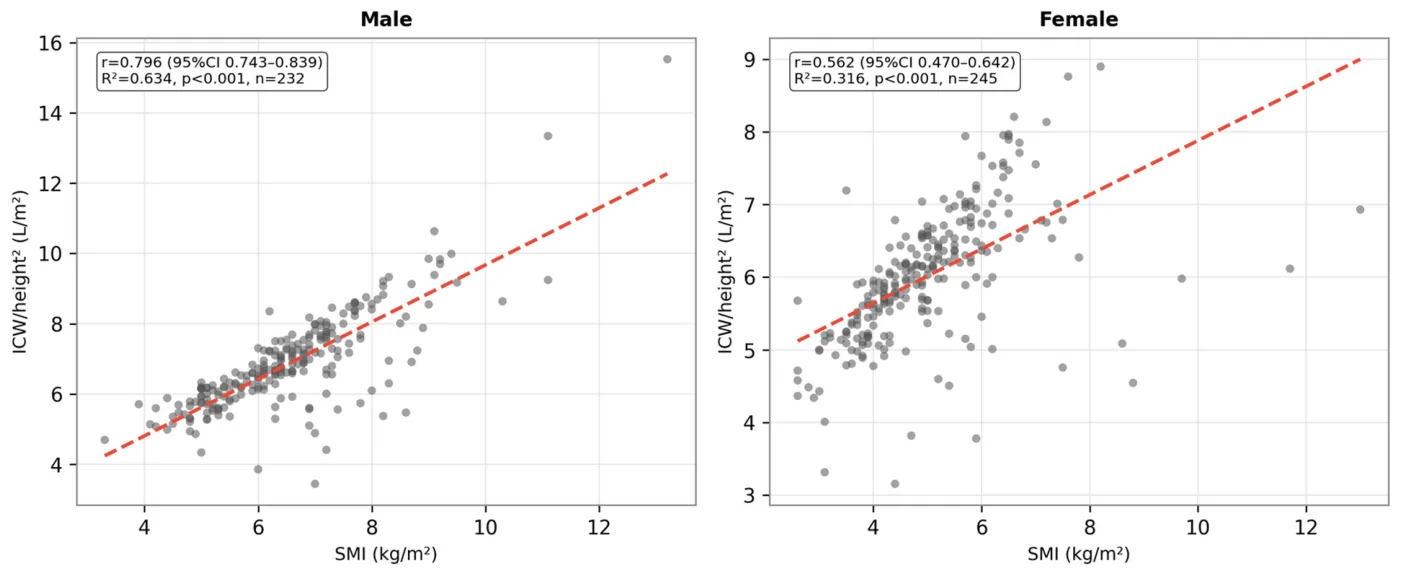
Relationship between SMI and ICW/height^2^. Scatter plots showing the Pearson correlation between SMI and ICW/height^2^ in men (**left**) and women (**right**). A strong correlation was observed in men (r = 0.796, 95% CI 0.743–0.839, R^2^ = 0.634, *p* < 0.001), whereas a weaker correlation was found in women (r = 0.562, 95% CI 0.470–0.642, R^2^ = 0.316, *p* < 0.001).

Overall, SMI and ICW/height^2^ were significantly correlated (r = 0.730, 95% CI 0.685–0.769, R^2^ = 0.532, *p* < 0.001). A strong positive correlation between SMI and ICW/height^2^ was observed in men (r = 0.796, 95% CI 0.743–0.839, R^2^ = 0.634, *p* < 0.001). In contrast, the correlation was substantially weaker in women (r = 0.562, 95% CI 0.470–0.642, R^2^ = 0.316, *p* < 0.001) (Figure 2). A formal Fisher’s z-test confirmed that the sex difference in correlation coefficients was statistically significant (z  =  4.90, *p*  <  0.001), indicating that the SMI–ICW/height^2^ relationship is significantly stronger in men than in women.

### 3.4. Association Between SMI and ICW/Height^2^ Across BMI Categories

**Figure 3:**
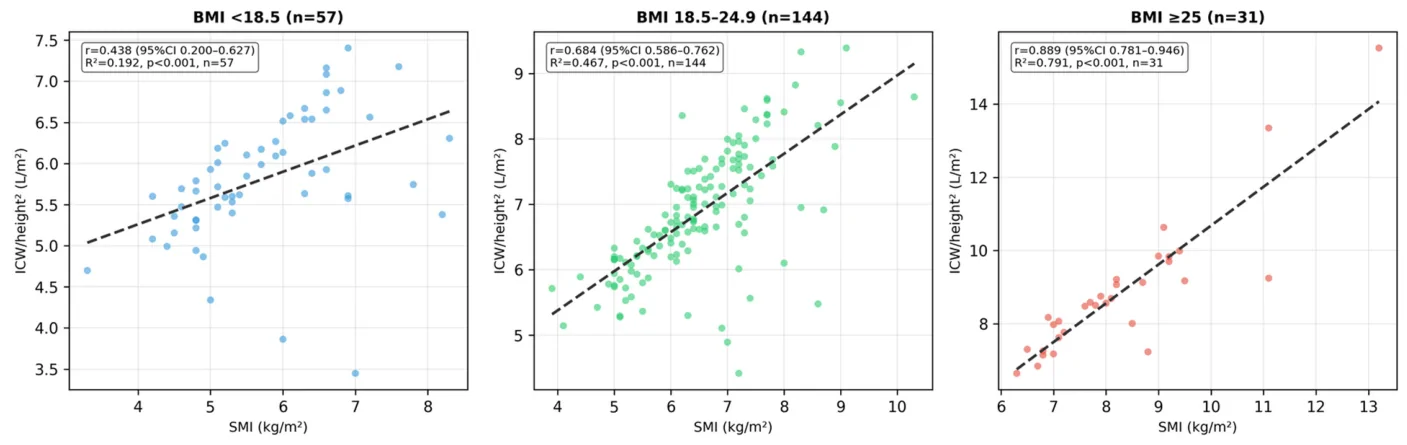
Association between SMI and ICW/height^2^ by BMI category in men. The correlation increased progressively with higher BMI: <18.5 (*n* = 57, R^2^ = 0.192, *p* < 0.001), 18.5–24.9 (*n* = 144, R^2^ = 0.467, *p* < 0.001), and ≥25 kg/m^2^ (*n* = 31, R^2^ = 0.791, *p* < 0.001). Pearson r with 95% CI and *p* values are shown in each panel.

**Figure 4:**
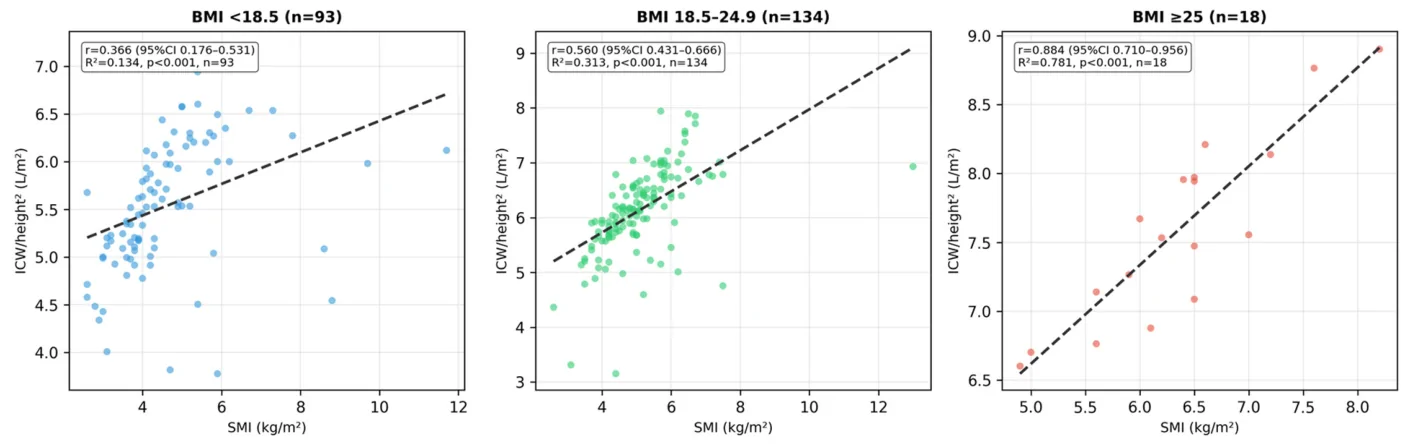
Association between SMI and ICW/height^2^ by BMI category in women. The correlation was markedly attenuated in the low-BMI group (<18.5: *n* = 93, R^2^ = 0.134, *p* < 0.001), remained moderate in the normal-BMI group (18.5–24.9: *n* = 134, R^2^ = 0.313, *p* < 0.001), and increased in the high-BMI group (≥25: *n* = 18, R^2^ = 0.781, *p* < 0.001).

Patients were stratified into three BMI categories: <18.5, 18.5–24.9, and ≥25 kg/m^2^. In men, the correlation between SMI and ICW/height^2^ increased progressively with higher BMI: BMI < 18.5 (*n* = 57): r = 0.438 (95% CI 0.200–0.627, R^2^ = 0.192, *p* < 0.001); BMI 18.5–24.9 (*n* = 144): r = 0.684 (95% CI 0.586–0.762, R^2^ = 0.467, *p* < 0.001); BMI ≥ 25 (*n* = 31): r = 0.889 (95% CI 0.781–0.946, R^2^ = 0.791, *p* < 0.001) (Figure 3). In women, the correlation was markedly attenuated in the low-BMI group: BMI < 18.5 (*n* = 93): r = 0.366 (95% CI 0.176–0.531, R^2^ = 0.134, *p* < 0.001); BMI 18.5–24.9 (*n* = 134): r = 0.560 (95% CI 0.431–0.666, R^2^ = 0.313, *p* < 0.001); BMI ≥ 25 (*n* = 18): r = 0.884 (95% CI 0.710–0.956, R^2^ = 0.781, *p* < 0.001) (Figure 4). Notably, in the low-BMI subgroup, the sex difference in correlation was especially pronounced (men R^2^ = 0.192 vs. women R^2^ = 0.134), suggesting a sex-specific pattern that is not explained by BMI alone.

### 3.5. Influence of Hydration Status (ECW/TBW)

**Figure 5:**
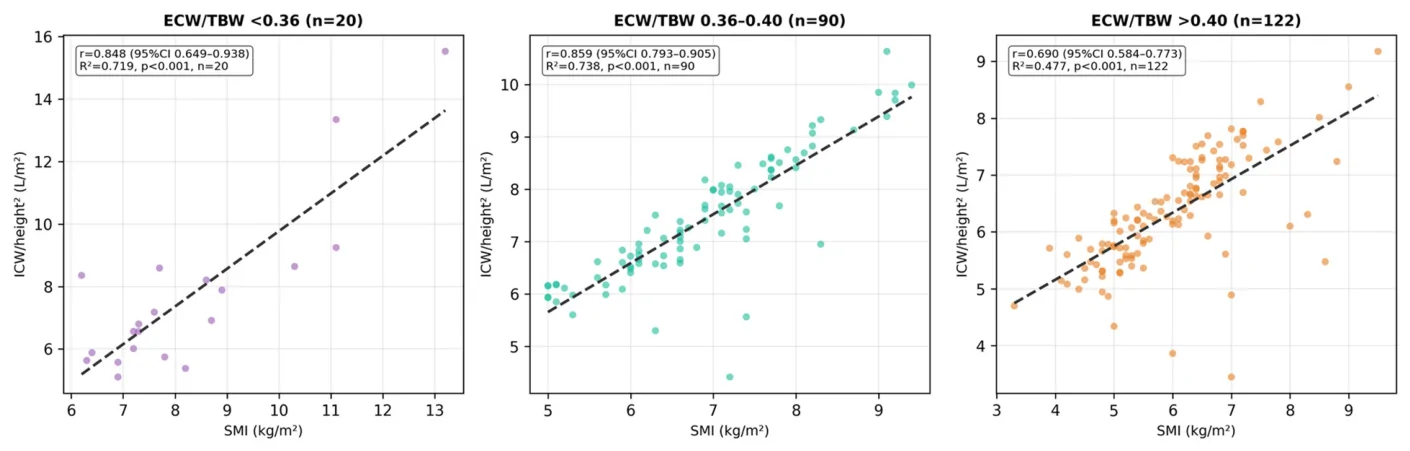
Association between SMI and ICW/height^2^ by ECW/TBW category in men. The correlation was strong in the low (<0.36: *n* = 20, R^2^ = 0.719) and normal ECW/TBW groups (0.36–0.40: *n* = 90, R^2^ = 0.738), but attenuated in the high ECW/TBW group (>0.40: *n* = 122, R^2^ = 0.477). All *p* < 0.001.

**Figure 6:**
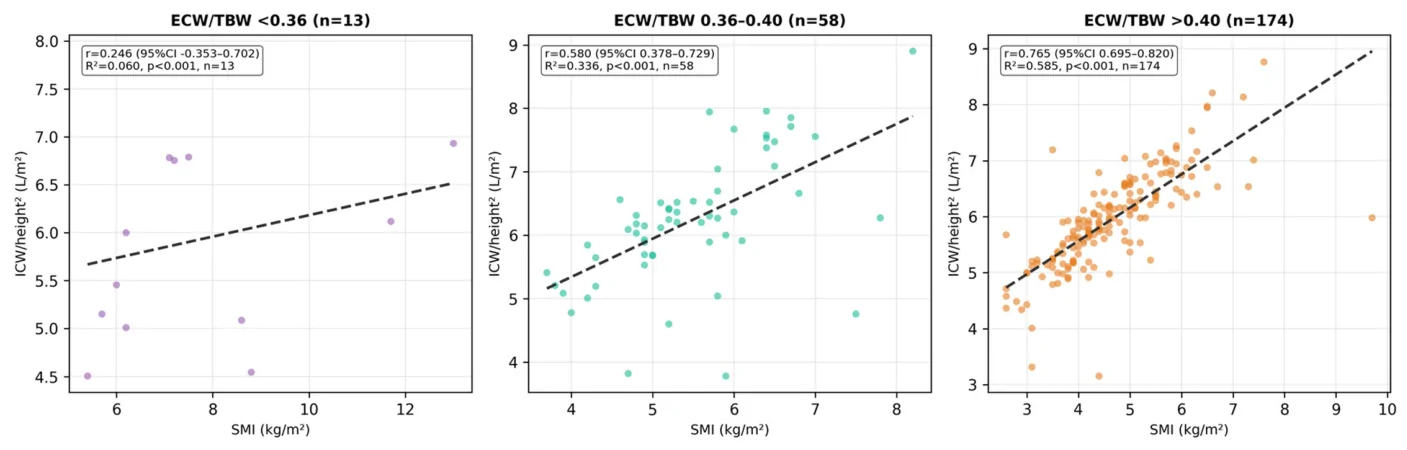
Association between SMI and ICW/height^2^ by ECW/TBW category in women. The correlation was negligible and non-significant in the low ECW/TBW group (<0.36: *n* = 13, r = 0.246, *p* = n.s.), remained modest in the normal range (0.36–0.40: *n* = 58, R^2^ = 0.336, *p* < 0.001), and was stronger in the high ECW/TBW group (>0.40: *n* = 174, R^2^ = 0.585, *p* < 0.001).

Patients were further stratified by ECW/TBW into three hydration categories: <0.36, 0.36–0.40, and >0.40. In men, the correlation between SMI and ICW/height^2^ was strong in both the low- and normal-hydration groups: ECW/TBW < 0.36 (*n* = 20): r = 0.848 (95% CI 0.649–0.938, R^2^ = 0.719, *p* < 0.001); ECW/TBW 0.36–0.40 (*n* = 90): r = 0.859 (95% CI 0.793–0.905, R^2^ = 0.738, *p* < 0.001). However, in men with high ECW/TBW (>0.40, *n* = 122), the correlation was attenuated: r = 0.690 (95% CI 0.584–0.773, R^2^ = 0.477, *p* < 0.001) (Figure 5). In women, the pattern differed markedly. The correlation was negligible and non-significant in the low ECW/TBW group (ECW/TBW < 0.36, *n* = 13): r = 0.246 (95% CI −0.353–0.702, R^2^ = 0.060, *p* = n.s.). Even within the normal ECW/TBW range (0.36–0.40, *n* = 58), the correlation remained modest: r = 0.580 (95% CI 0.378–0.729, R^2^ = 0.336, *p* < 0.001). A relatively stronger correlation was observed in women with high ECW/TBW (>0.40, *n* = 174): r = 0.765 (95% CI 0.695–0.820, R^2^ = 0.585, *p* < 0.001) (Figure 6).

### 3.6. Discordance Between SMI and ICW/Height^2^ in Women with Normal Hydration and Low BMI

Among women with normal ECW/TBW (0.36–0.40), 19 of 58 patients had BMI < 18.5 kg/m^2^. In this subgroup (*n* = 19), the correlation between SMI and ICW/height^2^ was weak and non-significant (r = 0.329, 95% CI −0.147–0.681, R^2^ = 0.108, *p* = n.s.), indicating clear discordance between the two indices despite normal hydration status. Although the small sample size precludes robust estimation, the pattern is consistent with the overall finding that ICW/height^2^ and SMI diverge in underweight women.

### 3.7. Multivariable Regression Analysis

Multivariable linear regression was performed with ICW/height^2^ as the dependent variable and SMI, BMI, ECW/TBW, and age as independent variables. In the overall model (*n* = 477), R^2^ = 0.672 (adjusted R^2^ = 0.668). SMI (β = 0.418, *p* < 0.001), BMI (β = 0.118, *p* < 0.001), and age (β = −0.010, *p* < 0.001) were significant independent predictors. ECW/TBW was also a significant predictor in the overall model (β = 3.44, *p* < 0.01). Sex was not a significant independent predictor (β = 0.019, *p* = n.s.), indicating that the sex-specific patterns observed in stratified analyses may reflect interactions rather than a direct effect of sex. In sex-stratified models, ECW/TBW was a significant predictor in women (β = 6.15, *p* < 0.001) but not in men (β = −0.21, *p* = n.s.), supporting a sex-specific relationship between hydration status and the SMI–ICW/height^2^ discordance. Adjusted R^2^ was higher in men (0.730) than in women (0.522), reflecting greater unexplained variability in ICW/height^2^ among women. To formally test the sex-specific effect of hydration status, a sex × ECW/TBW interaction term was added to the overall regression model. The interaction term was statistically significant (β = −12.258 L/m^2^ per unit ECW/TBW [i.e., approximately −0.12 L/m^2^ per 0.01-unit increment, or −0.36 L/m^2^ per one standard deviation (SD = 0.029) in this cohort], t = −5.59, *p* < 0.001), indicating that the effect of ECW/TBW on ICW/height^2^ was significantly stronger in women than in men. The interaction model achieved R^2^ = 0.692 (adjusted R^2^ = 0.688).

### 3.8. Results of Four-Quadrant Classification

**Figure 7:**
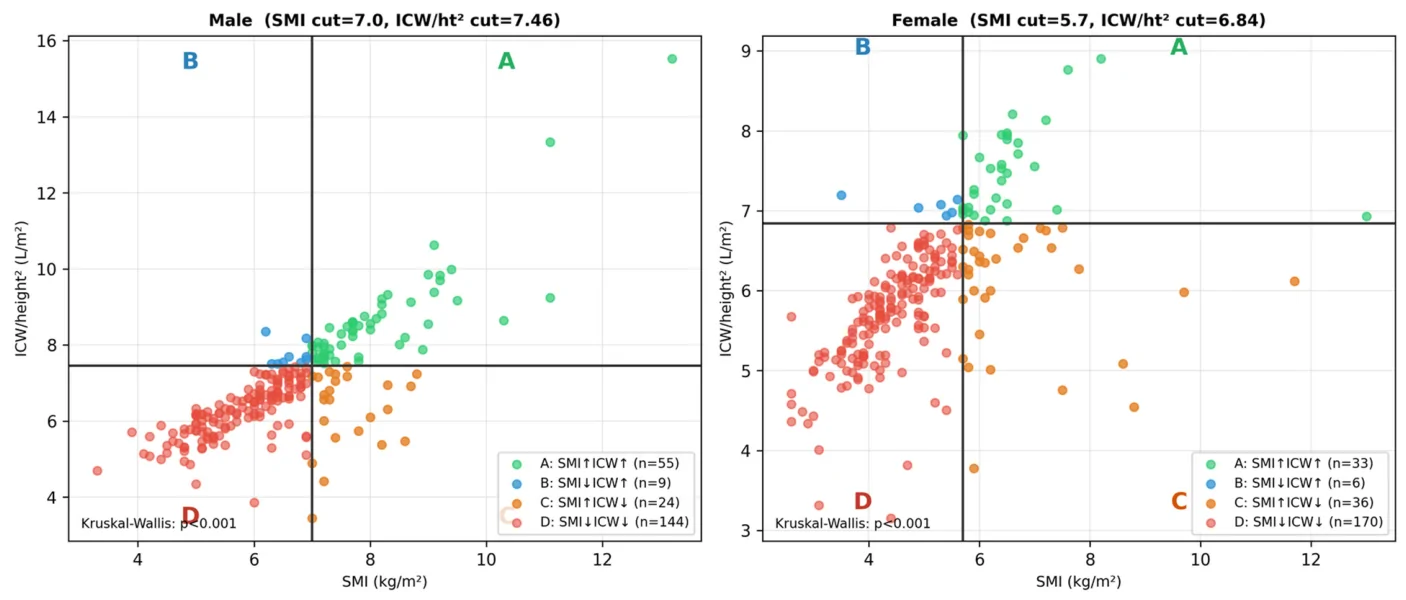
Exploratory four-quadrant classification based on sex-specific SMI and ICW/height^2^ thresholds. Patients were classified as A = SMI+/ICW+ (preserved both), B = SMI−/ICW+ (low SMI only), C = SMI+/ICW− (low ICW only), D = SMI−/ICW− (both low). Vertical/horizontal lines indicate cut-offs (men: SMI = 7.0 kg/m^2^, ICW/ht^2^ = 7.46 L/m^2^; women: SMI = 5.7 kg/m^2^, ICW/ht^2^ = 6.84 L/m^2^). BMI differed significantly across groups in both sexes (Kruskal–Wallis, *p* < 0.001).

For exploratory analysis, patients were classified into four groups using sex-specific cut-offs: SMI ≥ 7.0 kg/m^2^ (men) or ≥5.7 kg/m^2^ (women), and ICW/height^2^ ≥ 7.46 L/m^2^ (men) or ≥6.84 L/m^2^ (women) (these ICW/height^2^ thresholds were derived as median values from the present dataset and are considered provisional). Group A (SMI preserved, ICW/height^2^ preserved): men *n* = 55, BMI 25.3 ± 4.9, median 24.1 kg/m^2^; women *n* = 33, BMI 24.5 ± 3.7, median 24.4 kg/m^2^. Group B (SMI low, ICW/height^2^ preserved): men *n* = 9, women *n* = 6. Group C (SMI preserved, ICW/height^2^ low): men *n* = 24, BMI 20.4 ± 3.3; women *n* = 36, BMI 19.3 ± 3.0. Group D (both low): men *n* = 144, BMI 19.6 ± 3.1; women *n* = 170, BMI 18.9 ± 3.3. BMI differed significantly across the four groups in both men and women (Kruskal–Wallis, *p* < 0.001). Notably, Group C patients (preserved SMI with low ICW/height^2^) had lower BMI than Group A in both sexes, suggesting that ICW/height^2^ may help identify patients in whom BIA-derived SMI appears relatively preserved despite reduced intracellular body cell reserve (Figure 7). The Bland–Altman analysis confirmed that the limits of agreement between standardized SMI and ICW/height^2^ were wider in women (±1.834 SD) than in men (±1.252 SD), consistent with greater inter-index discordance in women (Supplementary Figure S1). Age differed significantly across the four groups (one-way ANOVA: F(3473)  =  22.57, *p*  <  0.001): Group A (69.6 ± 18.2 years), Group B (81.2 ± 12.4 years), Group C (77.7 ± 15.8 years), and Group D (82.6 ± 10.8 years). ECW/TBW also differed significantly across groups (Kruskal–Wallis: H  =  68.49, *p*  <  0.001): Group A (0.391 ± 0.032), Group B (0.405 ± 0.028), Group C (0.383 ± 0.046), and Group D (0.411 ± 0.021). Patients in Group A were the youngest with the lowest ECW/TBW, whereas those in Group D were the oldest with the highest ECW/TBW. Group B had a higher mean ECW/TBW (0.405) than Group A (0.391) and Group C (0.383), whereas Group C had the lowest mean ECW/TBW among the four groups (Table 1; further interpretation is provided in Section 4.4).

## 4. Discussion

### 4.1. Principal Findings

This retrospective study demonstrated that ICW/height^2^ and SMI, both derived from BIA, capture partially distinct aspects of body composition in hospitalized patients. While the two indices were significantly correlated overall, their relationship varied substantially by sex, BMI category, and hydration status. ICW/height^2^ showed stronger correlations with BMI than SMI in both sexes, and the discordance between SMI and ICW/height^2^ was most pronounced in women with low BMI. Multivariable regression confirmed that ECW/TBW was an independent predictor of ICW/height^2^ in women but not in men, supporting a sex-specific interaction between fluid status and the two indices. These findings suggest that ICW/height^2^ provides complementary information to SMI and may help interpret BIA-derived muscle assessment in patients with altered nutritional status or fluid distribution.

### 4.2. Hydration-Related Discordance Between SMI and ICW/Height^2^

A fundamental methodological consideration is that SMI, ICW/height^2^, and ECW/TBW are all derived from the same BIA measurement and share the same underlying raw impedance and anthropometric data; these variables are not statistically independent. The correlations and regression analyses should be interpreted within this constraint: the study does not claim independence between indices, but examines whether their relationship varies systematically across clinical subgroups.

BIA-derived SMI is known to be susceptible to fluid shifts. Extracellular fluid expansion may lead to potential overestimation of BIA-derived skeletal muscle mass estimates, because the impedance-based algorithm attributes increased total body water to lean mass. Consistent with this, the correlation between SMI and ICW/height^2^ was attenuated in men with ECW/TBW > 0.40 (R^2^ = 0.477) compared to those with normal ECW/TBW (R^2^ = 0.738). ICW/height^2^, by contrast, reflects water within the intracellular compartment and is less susceptible to extracellular fluid accumulation, which may make it a more stable indicator of intracellular body cell mass under conditions of fluid overload [4,5,9,12].

### 4.3. Sex-Specific and Nutritional Discordance, Particularly in Underweight Women

One possible mechanism, proposed here as a hypothesis consistent with prior literature rather than a direct conclusion of the present data, is that malnutrition—more prevalent in underweight women—may preferentially reduce intracellular volume (reflected by ICW/height^2^) while extracellular fluid is relatively preserved or even expanded. Under such conditions, BIA-derived SMI may remain relatively stable, leading to potential overestimation of BIA-derived skeletal muscle mass estimates. It should be emphasised that nutritional status was not directly measured in the present study using validated tools such as GLIM criteria [13], and disease severity and other potential confounders were not systematically controlled. This interpretation is therefore speculative and should be considered one of several possible explanations, rather than an established finding.

### 4.4. Exploratory Four-Quadrant Classification

The exploratory four-quadrant classification provided a practical framework for visualizing the discordance between SMI and ICW/height^2^. Patients with preserved SMI but low ICW/height^2^ (Group C) had lower BMI than those with both indices preserved (Group A) in both sexes, supporting the possibility that ICW/height^2^ may help identify patients in whom BIA-derived SMI appears relatively preserved despite reduced intracellular body cell reserve. The ICW/height^2^ thresholds used (7.46 L/m^2^ for men, 6.84 L/m^2^ for women) were derived as provisional cut-offs from the present dataset and require external validation before clinical application. Future studies comparing these thresholds against CT or DEXA-derived muscle mass will be needed to evaluate their clinical validity.

### 4.5. Clinical Implications

The present study demonstrates the existence of systematic discordance between BIA-derived SMI and ICW/height^2^ under specific clinical conditions. These are exploratory, hypothesis-generating findings and should not be interpreted as establishing clinical utility, validated diagnostic thresholds, or evidence of outcome benefit for ICW/height^2^. Before ICW/height^2^ can be considered for routine clinical application, prospective validation against reference-standard measurements (CT, MRI, or DEXA) and clinical outcome data is required.

ICW/height^2^ should not be interpreted as a replacement for SMI. Rather, it may serve as a complementary BIA-derived parameter that can support the interpretation of BIA findings in specific clinical contexts. In underweight hospitalized patients—particularly women—where SMI may appear relatively preserved but intracellular body cell volume is reduced, ICW/height^2^ may provide additional information for nutritional assessment. This may be especially relevant in settings where CT or DEXA are unavailable, and BIA is the primary tool for body composition evaluation. However, clinical implications should be interpreted cautiously, as this study did not assess outcomes such as muscle strength, physical performance, or nutritional status changes over time. This concept of complementary BIA-derived markers is also consistent with the recent shift from sarcopenia diagnosis alone toward broader muscle health assessment [14].

### 4.6. Limitations

This study has several limitations. First, this was a single-centre retrospective study, and the generalisability of the findings to other populations and settings requires validation. Second, SMI and ICW/height^2^ were not validated against reference methods such as CT, MRI, or DEXA; therefore, we cannot determine which index more accurately reflects reference-standard muscle mass or intracellular body cell mass. Third, the ICW/height^2^ thresholds used in the four-quadrant classification were derived from the present dataset and should be regarded as exploratory and hypothesis-generating; a split-sample validation was not performed due to sample size constraints, and external validation is required. Fourth, the cross-sectional design precludes conclusions about longitudinal changes or clinical outcomes. Fifth, we could not account for disease severity, medication use, or time since admission, which may independently affect body composition. Sixth, body composition measurements were performed exclusively using the InBody S10 in the supine position; a single device was available at the institution, and all measurements followed the same standardised protocol. Findings may therefore not be directly comparable to studies using standing BIA devices or different manufacturers. Seventh, the small sample sizes in some subgroups (e.g., women with ECW/TBW < 0.36, *n* = 13; women with BMI ≥ 25, *n* = 18; women with normal ECW/TBW and low BMI, *n* = 19) limit the precision of correlation estimates and warrant cautious interpretation. Eighth, formal malnutrition diagnosis using GLIM criteria [13] or nutritional risk scoring was not available; BMI was used as the primary nutritional stratification variable, acknowledging its limitations in hospitalised patients with fluid imbalance. Ninth, SMI, ICW/height^2^, and ECW/TBW are derived from the same BIA measurement and are not independent variables; this limits causal inference from the correlation and regression analyses. Tenth, the multiple subgroup analyses (stratified by BMI category, ECW/TBW category, and sex) were performed without correction for multiple comparisons. All subgroup findings should therefore be interpreted as exploratory and hypothesis-generating; formal correction for multiple comparisons (e.g., Bonferroni or false discovery rate) would be appropriate in future confirmatory studies.

## Figures and Tables

**Table 1:** Patient characteristics.

Variable	All (*n* = 477)	Male (*n* = 232)	Female (*n* = 245)	*p* Value
Age (years)	79.5 ± 14.1	76.0 ± 14.1	82.9 ± 13.2	<0.001
Height (cm)	156.4 ± 10.1	163.9 ± 7.3	149.3 ± 6.6	<0.001
Weight (kg)	50.4 ± 13.2	56.9 ± 13.2	44.2 ± 9.8	<0.001
BMI (kg/m^2^)	20.4 ± 4.1	21.1 ± 4.3	19.8 ± 3.8	0.002
SMI (kg/m^2^)	5.8 ± 1.6	6.5 ± 1.4	5.0 ± 1.4	<0.001
ICW/height^2^ (L/m^2^)	6.4 ± 1.2	6.9 ± 1.4	6.0 ± 0.9	<0.001
ECW/TBW	0.404 ± 0.029	0.398 ± 0.028	0.409 ± 0.030	<0.001
Albumin (g/dL)	3.6 ± 1.1	3.6 ± 1.0	3.6 ± 1.2	0.789
Phase angle (°)	4.5 ± 2.1	5.0 ± 2.2	3.9 ± 1.9	<0.001
Low SMI, *n* (%)	329 (69.0%)	153 (65.9%)	176 (71.8%)	0.155
BMI < 18.5, *n* (%)	150 (31.4%)	57 (24.6%)	93 (38.0%)	<0.001
BMI 18.5–24.9, *n* (%)	278 (58.3%)	144 (62.1%)	134 (54.7%)	0.094
BMI ≥ 25, *n* (%)	49 (10.3%)	31 (13.4%)	18 (7.3%)	0.037
ECW/TBW < 0.36, *n* (%)	33 (6.9%)	20 (8.6%)	13 (5.3%)	0.141
ECW/TBW 0.36–0.40, *n* (%)	148 (31.0%)	90 (38.8%)	58 (23.7%)	<0.001
ECW/TBW > 0.40, *n* (%)	296 (62.1%)	122 (52.6%)	174 (71.0%)	<0.001
Barthel Index at admission	60 (5–95)	65 (5–100)	55 (2–90)	0.088
Length of stay (days)	27 (11–47)	28 (12–46)	27 (11–50)	0.799
Disease group, *n* (%)				<0.01
Internal medicine	138 (28.9%)	54 (23.3%)	84 (34.3%)	
Cerebrovascular	122 (25.6%)	57 (24.6%)	65 (26.5%)	
Respiratory	68 (14.3%)	46 (19.8%)	22 (9.0%)	
Cancer	37 (7.8%)	26 (11.2%)	11 (4.5%)	
Musculoskeletal	35 (7.3%)	17 (7.3%)	18 (7.3%)	
Urology	29 (6.1%)	12 (5.2%)	17 (6.9%)	
Cardiovascular	25 (5.2%)	9 (3.9%)	16 (6.5%)	
Disuse syndrome	23 (4.8%)	11 (4.7%)	12 (4.9%)	
Admission ward, *n* (%)				0.380
Ward 3 (rehabilitation)	196 (41.1%)	96 (41.4%)	100 (40.8%)	
Ward 4 (internal medicine/respiratory)	260 (54.5%)	128 (55.2%)	132 (53.9%)	
Other wards	21 (4.4%)	8 (3.4%)	13 (5.3%)	

Data are expressed as mean ± standard deviation, median (interquartile range), or *n* (%). *p* values for sex comparison were derived using Student’s *t*-test (continuous variables) or chi-square test (categorical variables). BMI, body mass index; SMI, skeletal muscle index; ICW, intracellular water; ECW/TBW, extracellular water-to-total body water ratio.

## Data Availability

The data presented in this study are available on request from the corresponding author due to privacy and ethical restrictions.

## Supplementary Materials

The following supporting information can be downloaded at: https://www.mdpi.com/article/10.3390/muscles5030054/s1, Figure S1. Bland–Altman plots of agreement between standardised skeletal muscle index (SMI) and ICW/height^2^ in men and women. Data points are standardized (z-score) for cross-comparison. The solid line indicates the mean difference (bias) and the dashed lines indicate the limits of agreement (±1.96 SD). Data are stratified by sex: men (upper) and women (lower). ICW, intracellular water; SMI, skeletal muscle index.
