# Supplementary material for: Intracellular Water Indexed to Height Squared as a Complementary Marker to Skeletal Muscle Index Across Nutritional and Hydration Status
Source: Muscles. 2026 Jul 29;5(3):54. doi: 10.3390/muscles5030054 (PMC13510820; doi:10.3390/muscles5030054)
Supplement: Supplementary file 1 [file muscles-05-00054-s001.zip › muscles-4398347-supplementary.pdf]

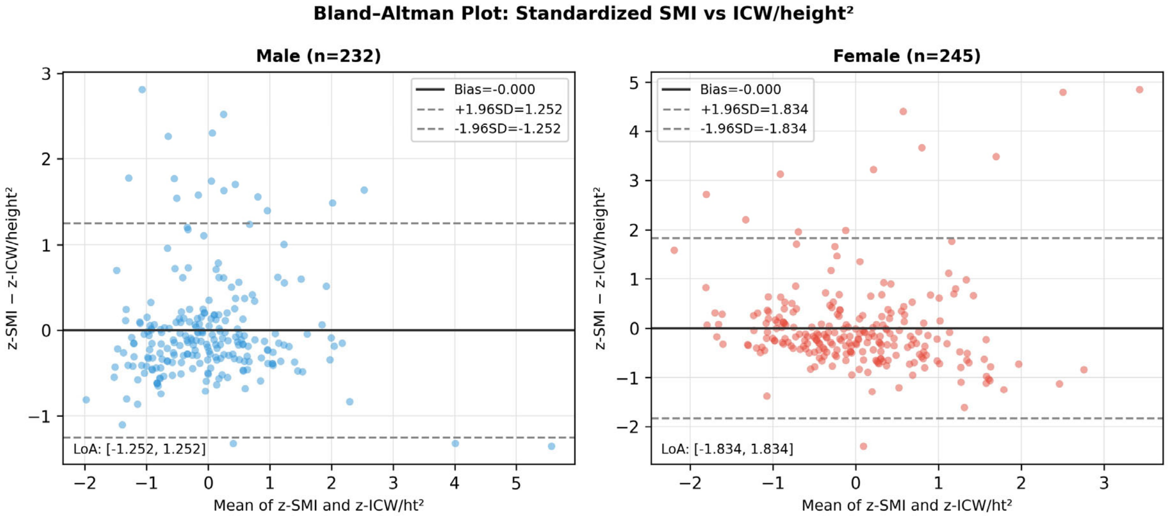

Figure S1. Bland–Altman plots of agreement between standardised skeletal muscle index (SMI) and ICW/height² in men and women. Data points are standardized (z-score) for cross-comparison. The solid line indicates the mean difference (bias) and the dashed lines indi-cate the limits of agreement (±1.96 SD). Data are stratified by sex: men (upper) and women (low-er). ICW, intracellular water; SMI, skeletal muscle index.
